# Bis(*n*-do­decyl­ammonium) bis­(chlor­anil­ato)di­ethano­lcuprate(II)

**DOI:** 10.1107/S1600536814001202

**Published:** 2014-01-22

**Authors:** Akiko Himegi, Satoshi Kawata

**Affiliations:** a Department of Chemistry, Faculty of Science, Fukuoka University, Nanakuma, Jonan-ku, Fukuoka 814-0180, Japan

## Abstract

In the title compound, (C_12_H_25_NH_3_)_2_[Cu(C_6_Cl_2_O_4_)_2_(C_2_H_5_OH)_2_], the Cu^II^ atom lies on a crystallographic inversion center and is coordinated in a distorted octa­hedral geometry by four O atoms of two chloranilate ligands and two O atoms of two ethanol mol­ecules which are *trans* to each other in the axial positions. In the crystal, the Cu^II^ mononuclear dianions are linked by O—H⋯O hydrogen bonds into a tape along the *a-*axis direction. The tapes are linked through N—H⋯O hydrogen bonds between the dianion and the *n*-do­decyl­ammonium cation, forming a two-dimensional network parallel to the *ab* plane.

## Related literature   

For metal complexes of chloranilic acid, see: Kawata & Kitagawa (2002 ▶); Kawata *et al.* (2000 ▶); Luo *et al.* (2004 ▶); Abrahams *et al.* (2011 ▶); Nagayoshi *et al.* (2003 ▶); Nishimura *et al.* (2013 ▶).
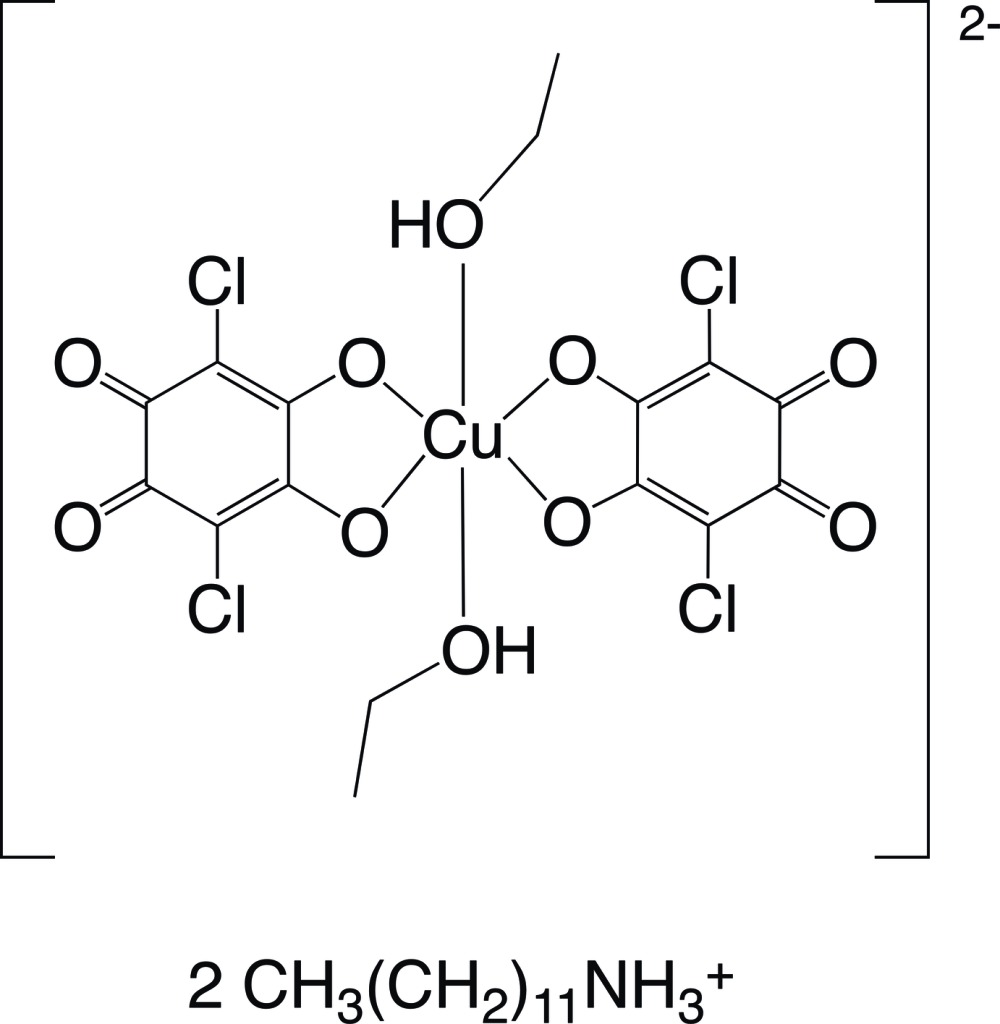


## Experimental   

### 

#### Crystal data  

(C_12_H_28_N)_2_[Cu(C_6_Cl_2_O_4_)_2_(C_2_H_6_O)_2_]
*M* *_r_* = 942.34
Triclinic, 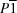

*a* = 9.2192 (15) Å
*b* = 9.4791 (13) Å
*c* = 15.162 (3) Å
α = 76.894 (9)°
β = 89.133 (10)°
γ = 63.110 (6)°
*V* = 1145.1 (4) Å^3^
*Z* = 1
Mo *K*α radiation
μ = 0.76 mm^−1^
*T* = 100 K
0.40 × 0.30 × 0.05 mm

#### Data collection  

Rigaku Saturn724 diffractometer
Absorption correction: multi-scan (*REQAB*; Rigaku, 1998 ▶) *T*_min_ = 0.868, *T*_max_ = 0.962
17151 measured reflections
5207 independent reflections
4796 reflections with *I* > 2σ(*I*)
*R*_int_ = 0.025

#### Refinement  

*R*[*F* ^2^ > 2σ(*F* ^2^)] = 0.031
*wR*(*F* ^2^) = 0.079
*S* = 1.09
5207 reflections
275 parameters
H atoms treated by a mixture of independent and constrained refinement
Δρ_max_ = 0.92 e Å^−3^
Δρ_min_ = −0.34 e Å^−3^

### 

Data collection: *CrystalClear* (Rigaku, 2010 ▶); cell refinement: *CrystalClear*; data reduction: *CrystalClear*; program(s) used to solve structure: *Il Milione* (Burla *et al.*, 2007 ▶); program(s) used to refine structure: *SHELXL97* (Sheldrick, 2008 ▶); molecular graphics: *CrystalStructure* (Rigaku, 2010 ▶); software used to prepare material for publication: *CrystalStructure*.

## Supplementary Material

Crystal structure: contains datablock(s) General, I. DOI: 10.1107/S1600536814001202/is5334sup1.cif

Structure factors: contains datablock(s) I. DOI: 10.1107/S1600536814001202/is5334Isup2.hkl

CCDC reference:

Additional supporting information:  crystallographic information; 3D view; checkCIF report

## Figures and Tables

**Table 1:** Selected bond lengths (Å)

Cu1—O1	1.9489 (10)
Cu1—O2	1.9657 (10)
Cu1—O5	2.4097 (13)

**Table 2:** Hydrogen-bond geometry (Å, °)

*D*—H⋯*A*	*D*—H	H⋯*A*	*D*⋯*A*	*D*—H⋯*A*
O5—H1⋯O3^i^	0.88 (3)	1.94 (3)	2.8145 (16)	172 (3)
N1—H2⋯O1^ii^	0.91 (3)	1.97 (2)	2.8562 (16)	167 (3)
N1—H3⋯O3	0.89 (3)	2.05 (3)	2.928 (2)	168.3 (17)
N1—H4⋯O3^iii^	0.87 (3)	2.12 (3)	2.9784 (19)	171 (3)
N1—H4⋯O4^iii^	0.87 (3)	2.50 (3)	2.9842 (19)	116.2 (13)

Symmetry codes: (i) 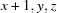; (ii) 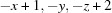; (iii) 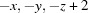.

**Fig. 1.**
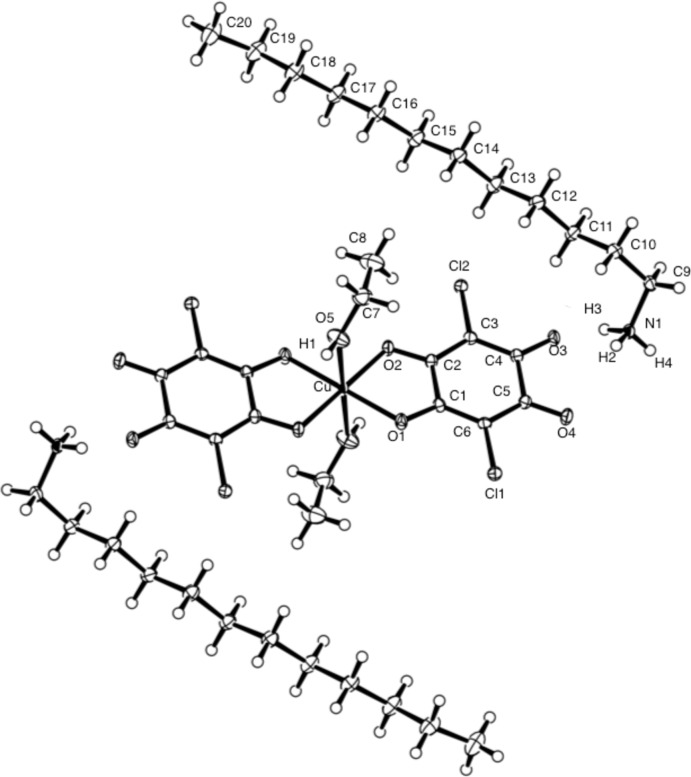
An ORTEP drawing of the title complex compound, showing 50% probability displacement elipsoids.

**Fig. 2.**
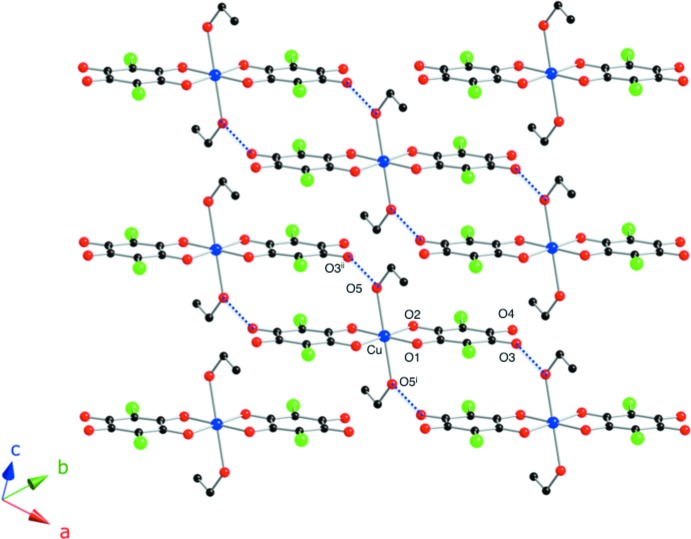
A packing diagram showing tape structures of [Cu(CA)2(EtOH)2]2- ions. The dashed lines denote the O—H···O hydrogen bonds.

**Fig. 3.**
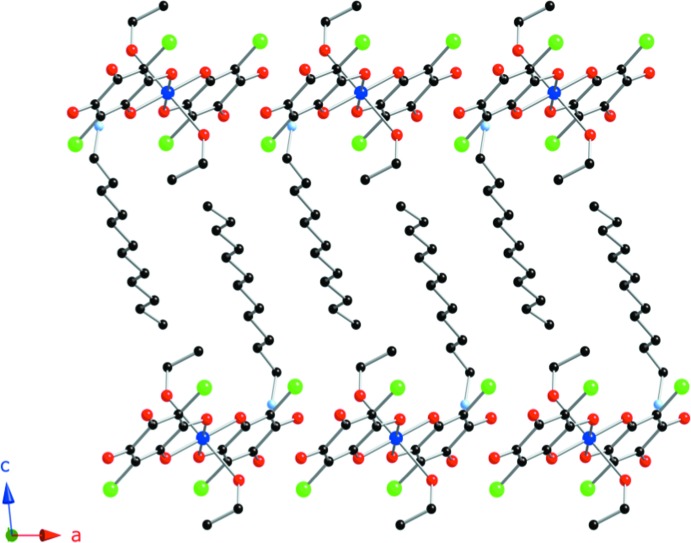
A packing diagram of the title compound, viewed along the b axis.

## supplementary crystallographic information

### 1. Comment

Chloranilic acid (H_2_CA = 2,5-dichloro-3,6-dihydroxy-1,4-benzoquinone) and its homologues, which contain two chelating coordination sites, are capable of bridging metal centers to form monomeric molecules, chains, sheets, or three-dimensional structures (Kawata & Kitagawa, 2002; Luo *et al.*, 2004; Abrahams *et al.*, 2011). In line with our study of metal-chloranilate complexes, we have been trying to develop metal-chloranilate hybrid materials and have found host–guest compounds (Kawata *et al.*, 2000; Nagayoshi *et al.*, 2003; Nishimura *et al*., 2013). We report here, a novel inorganic-organic hybrid system by using metal-chloranirate complexes as host layers and alkylamines as guests.

The title compound, (C_12_H_25_NH_3_)_2_[Cu(C_6_Cl_2_O_4_)_2_(C_2_H_5_OH)_2_], consists of the mononuclear [Cu(CA)_2_(EtOH)_2_]^2-^ dianion and the protonated *n*-dodecylamine (Hda^+^). The Cu^II^ atom lies on a crystallographic inversion center. The geometry around the Cu^II^ atom is a distorted octahedron involving four O atoms of two CA^2-^ anions and two O atoms from two ethanol molecules which are *trans* to each other. The axial bond distances are much longer than the equatorial ones (Table 1). The [Cu(CA)_2_(EtOH)_2_]^2-^ anions make a tape structure running along the *a* axis (Fig. 2) *via* an intermolecular O—H···O hydrogen bond (Table 2) between the coordinated ethanol molecule and the terminal oxygen atom of CA^2-^. The tapes are linked through N—H···O hydrogen bonds (Table 2) between the dianion and the Hda^+^ cation, forming a two-dimensional network expanding parallel to the *ab* plane (Fig. 3).

### 2. Experimental

An aqueous solution of copper sulfate pentahydrate (1 ml, 20 mmol *L*^-1^) was transferred to a glass tube, and then a mixture of *n*-dodecylamine (1 ml, 40 mmol *L*^-1^) in ethanol-water (1:1) solution and H_2_CA (1 ml, 60 mmol *L*^-1^) in ethanol solution was poured into the tube without mixing the two solutions. Violet crystals began to form at ambient temperature within two weeks. One of these crystals was used for X-ray crystallography.

### 3. Refinement

The C-bound H atoms in the alkyl chain of Hda^+^ ion and the ethyl group of the ethanol molecule were placed at calculated positions with C—H = 0.99 (CH_2_) and C—H = 0.98 (CH_3_) Å, and were treated as riding on their parent atoms with *U*_iso_(H) set to 1.2*U*_eq_(C). The O-bound and N-bound H atoms were located in a difference Fourier map and refined freely.

### Crystal data

**Table:**

(C_12_H_28_N)_2_[Cu(C_6_Cl_2_O_4_)_2_(C_2_H_6_O)_2_]	*Z* = 1
*M**_r_* = 942.34	*F*(000) = 499.00
Triclinic, *P*1	*D*_x_ = 1.366 Mg m^−^^3^
Hall symbol: -P 1	Mo *K*α radiation, λ = 0.71075 Å
*a* = 9.2192 (15) Å	Cell parameters from 3320 reflections
*b* = 9.4791 (13) Å	θ = 3.1–27.5°
*c* = 15.162 (3) Å	µ = 0.76 mm^−^^1^
α = 76.894 (9)°	*T* = 100 K
β = 89.133 (10)°	Platelet, violet
γ = 63.110 (6)°	0.40 × 0.30 × 0.05 mm
*V* = 1145.1 (4) Å^3^	

### Data collection

**Table:**

Rigaku Saturn724 diffractometer	4796 reflections with *F*^2^ > 2.0σ(*F*^2^)
Detector resolution: 7.111 pixels mm^-1^	*R*_int_ = 0.025
ω scans	θ_max_ = 27.5°
Absorption correction: multi-scan (*REQAB*; Rigaku, 1998)	*h* = −11→11
*T*_min_ = 0.868, *T*_max_ = 0.962	*k* = −12→12
17151 measured reflections	*l* = −19→19
5207 independent reflections	

### Refinement

**Table:**

Refinement on *F*^2^	Secondary atom site location: difference Fourier map
*R*[*F*^2^ > 2σ(*F*^2^)] = 0.031	Hydrogen site location: inferred from neighbouring sites
*wR*(*F*^2^) = 0.079	H atoms treated by a mixture of independent and constrained refinement
*S* = 1.09	*w* = 1/[σ^2^(*F*_o_^2^) + (0.037*P*)^2^ + 0.6543*P*] where *P* = (*F*_o_^2^ + 2*F*_c_^2^)/3
5207 reflections	(Δ/σ)_max_ = 0.001
275 parameters	Δρ_max_ = 0.92 e Å^−^^3^
0 restraints	Δρ_min_ = −0.34 e Å^−^^3^
Primary atom site location: structure-invariant direct methods	

### Special details

**Table:**

Geometry. ENTER SPECIAL DETAILS OF THE MOLECULAR GEOMETRY
Refinement. Refinement was performed using all reflections. The weighted *R*-factor (*wR*) and goodness of fit (*S*) are based on *F*^2^. *R*-factor (gt) are based on *F*. The threshold expression of *F*^2^ > 2.0 σ(*F*^2^) is used only for calculating *R*-factor (gt).

### Fractional atomic coordinates and isotropic or equivalent isotropic displacement parameters (Å^2^)

**Table:**

	*x*	*y*	*z*	*U*_iso_*/*U*_eq_	
Cu1	0.5000	0.5000	1.0000	0.01636 (8)	
Cl1	0.53483 (4)	−0.04086 (4)	1.14542 (2)	0.01736 (8)	
Cl2	−0.00556 (4)	0.55604 (4)	0.85100 (2)	0.01622 (8)	
O1	0.52991 (12)	0.28029 (12)	1.05403 (7)	0.0178 (2)	
O2	0.29500 (12)	0.53156 (12)	0.94162 (7)	0.0165 (2)	
O3	−0.00228 (12)	0.23827 (12)	0.94210 (7)	0.0169 (2)	
O4	0.22249 (13)	−0.00706 (13)	1.06624 (7)	0.0198 (2)	
O5	0.66424 (15)	0.42290 (16)	0.87749 (8)	0.0302 (3)	
N1	0.12545 (15)	−0.08274 (16)	0.90253 (9)	0.0157 (2)	
C1	0.40568 (16)	0.26123 (17)	1.03371 (9)	0.0135 (3)	
C2	0.27283 (16)	0.40582 (16)	0.96559 (9)	0.0135 (3)	
C3	0.13943 (16)	0.39417 (17)	0.93232 (9)	0.0139 (3)	
C4	0.11687 (16)	0.25682 (16)	0.96583 (9)	0.0135 (3)	
C5	0.24721 (16)	0.11209 (16)	1.03867 (9)	0.0139 (3)	
C6	0.38592 (17)	0.12498 (17)	1.06779 (9)	0.0141 (3)	
C7	0.6455 (2)	0.3845 (2)	0.79473 (12)	0.0291 (4)	
H7A	0.6655	0.2697	0.8072	0.035*	
H7B	0.7267	0.3966	0.7543	0.035*	
C8	0.4764 (2)	0.4956 (3)	0.74834 (13)	0.0372 (4)	
H8A	0.4575	0.6090	0.7352	0.045*	
H8B	0.3963	0.4826	0.7882	0.045*	
H8C	0.4648	0.4678	0.6913	0.045*	
C9	0.08326 (18)	−0.09264 (18)	0.80967 (10)	0.0191 (3)	
H9A	−0.0364	−0.0289	0.7937	0.023*	
H9B	0.1153	−0.2077	0.8105	0.023*	
C10	0.16933 (18)	−0.02718 (18)	0.73788 (10)	0.0190 (3)	
H10A	0.1583	−0.0583	0.6812	0.023*	
H10B	0.2873	−0.0805	0.7590	0.023*	
C11	0.10505 (18)	0.15795 (18)	0.71583 (10)	0.0192 (3)	
H11A	−0.0115	0.2123	0.6917	0.023*	
H11B	0.1121	0.1906	0.7726	0.023*	
C12	0.20052 (18)	0.21605 (19)	0.64636 (10)	0.0198 (3)	
H12A	0.3173	0.1602	0.6704	0.024*	
H12B	0.1925	0.1840	0.5896	0.024*	
C13	0.13993 (19)	0.40005 (19)	0.62405 (11)	0.0220 (3)	
H13A	0.1439	0.4327	0.6812	0.026*	
H13B	0.0244	0.4558	0.5979	0.026*	
C14	0.23900 (19)	0.45781 (19)	0.55735 (11)	0.0212 (3)	
H14A	0.3548	0.4015	0.5832	0.025*	
H14B	0.2340	0.4264	0.4999	0.025*	
C15	0.17839 (19)	0.64171 (19)	0.53626 (11)	0.0222 (3)	
H15A	0.1724	0.6741	0.5944	0.027*	
H15B	0.0662	0.6972	0.5051	0.027*	
C16	0.28427 (19)	0.70182 (19)	0.47717 (11)	0.0236 (3)	
H16A	0.2956	0.6647	0.4203	0.028*	
H16B	0.3948	0.6522	0.5098	0.028*	
C17	0.2144 (2)	0.88713 (19)	0.45306 (11)	0.0243 (3)	
H17A	0.1068	0.9357	0.4173	0.029*	
H17B	0.1959	0.9241	0.5102	0.029*	
C18	0.3209 (2)	0.9526 (2)	0.39921 (12)	0.0273 (4)	
H18A	0.3426	0.9137	0.3427	0.033*	
H18B	0.4272	0.9083	0.4356	0.033*	
C19	0.2427 (2)	1.1383 (2)	0.37427 (13)	0.0307 (4)	
H19A	0.1422	1.1817	0.3327	0.037*	
H19B	0.2100	1.1772	0.4302	0.037*	
C20	0.3529 (3)	1.2073 (2)	0.32906 (15)	0.0389 (5)	
H20A	0.3835	1.1722	0.2726	0.047*	
H20B	0.4516	1.1675	0.3703	0.047*	
H20C	0.2946	1.3268	0.3151	0.047*	
H1	0.769 (3)	0.374 (3)	0.8970 (16)	0.044 (6)*	
H2	0.235 (3)	−0.131 (2)	0.9169 (13)	0.021 (5)*	
H3	0.088 (2)	0.020 (2)	0.9063 (12)	0.020 (4)*	
H4	0.082 (2)	−0.129 (2)	0.9432 (14)	0.025 (5)*	

### Atomic displacement parameters (Å^2^)

**Table:**

	*U*^11^	*U*^22^	*U*^33^	*U*^12^	*U*^13^	*U*^23^
Cu1	0.01280 (12)	0.01202 (12)	0.02484 (14)	−0.00770 (10)	−0.00397 (9)	−0.00092 (10)
Cl1	0.01591 (16)	0.01415 (16)	0.01890 (16)	−0.00615 (13)	−0.00402 (12)	0.00037 (12)
Cl2	0.01428 (15)	0.01421 (16)	0.01823 (16)	−0.00668 (12)	−0.00354 (12)	0.00018 (12)
O1	0.0131 (5)	0.0140 (5)	0.0256 (6)	−0.0073 (4)	−0.0046 (4)	−0.0007 (4)
O2	0.0147 (5)	0.0131 (5)	0.0222 (5)	−0.0083 (4)	−0.0027 (4)	−0.0008 (4)
O3	0.0140 (5)	0.0160 (5)	0.0223 (5)	−0.0092 (4)	−0.0012 (4)	−0.0024 (4)
O4	0.0184 (5)	0.0163 (5)	0.0257 (6)	−0.0116 (5)	−0.0023 (4)	0.0013 (5)
O5	0.0188 (6)	0.0397 (7)	0.0288 (7)	−0.0085 (6)	−0.0005 (5)	−0.0129 (6)
N1	0.0147 (6)	0.0150 (6)	0.0187 (6)	−0.0089 (5)	0.0003 (5)	−0.0017 (5)
C1	0.0107 (6)	0.0137 (7)	0.0164 (7)	−0.0055 (5)	0.0006 (5)	−0.0045 (5)
C2	0.0121 (6)	0.0126 (7)	0.0164 (7)	−0.0059 (5)	0.0020 (5)	−0.0041 (5)
C3	0.0112 (6)	0.0131 (7)	0.0157 (7)	−0.0046 (5)	−0.0007 (5)	−0.0024 (5)
C4	0.0115 (6)	0.0136 (7)	0.0159 (7)	−0.0058 (5)	0.0019 (5)	−0.0046 (5)
C5	0.0131 (7)	0.0134 (7)	0.0153 (7)	−0.0065 (6)	0.0021 (5)	−0.0032 (5)
C6	0.0128 (6)	0.0126 (6)	0.0146 (6)	−0.0046 (5)	−0.0019 (5)	−0.0015 (5)
C7	0.0311 (9)	0.0265 (9)	0.0253 (8)	−0.0106 (8)	−0.0011 (7)	−0.0038 (7)
C8	0.0294 (10)	0.0532 (12)	0.0259 (9)	−0.0172 (9)	−0.0008 (7)	−0.0082 (9)
C9	0.0191 (7)	0.0196 (7)	0.0211 (7)	−0.0118 (6)	−0.0029 (6)	−0.0032 (6)
C10	0.0194 (7)	0.0191 (7)	0.0184 (7)	−0.0091 (6)	0.0018 (6)	−0.0040 (6)
C11	0.0185 (7)	0.0187 (7)	0.0190 (7)	−0.0090 (6)	0.0017 (6)	−0.0009 (6)
C12	0.0192 (7)	0.0216 (8)	0.0168 (7)	−0.0093 (6)	0.0010 (6)	−0.0014 (6)
C13	0.0196 (7)	0.0219 (8)	0.0231 (8)	−0.0100 (6)	0.0042 (6)	−0.0020 (6)
C14	0.0209 (8)	0.0220 (8)	0.0197 (7)	−0.0104 (6)	0.0026 (6)	−0.0022 (6)
C15	0.0180 (7)	0.0215 (8)	0.0241 (8)	−0.0084 (6)	0.0030 (6)	−0.0017 (6)
C16	0.0211 (8)	0.0207 (8)	0.0253 (8)	−0.0085 (6)	0.0053 (6)	−0.0012 (6)
C17	0.0221 (8)	0.0215 (8)	0.0249 (8)	−0.0083 (7)	0.0062 (6)	−0.0016 (6)
C18	0.0256 (8)	0.0215 (8)	0.0305 (9)	−0.0092 (7)	0.0098 (7)	−0.0027 (7)
C19	0.0348 (10)	0.0216 (9)	0.0308 (9)	−0.0105 (8)	0.0100 (8)	−0.0033 (7)
C20	0.0473 (12)	0.0270 (10)	0.0437 (11)	−0.0195 (9)	0.0166 (9)	−0.0067 (8)

### Geometric parameters (Å, º)

**Table:**

Cu1—O1^i^	1.9489 (11)	C10—C11	1.530 (2)
Cu1—O1	1.9489 (10)	C10—H10A	0.9900
Cu1—O2^i^	1.9657 (10)	C10—H10B	0.9900
Cu1—O2	1.9657 (10)	C11—C12	1.528 (2)
Cu1—O5^i^	2.4097 (13)	C11—H11A	0.9900
Cu1—O5	2.4097 (13)	C11—H11B	0.9900
Cl1—C6	1.7317 (14)	C12—C13	1.525 (2)
Cl2—C3	1.7315 (14)	C12—H12A	0.9900
O1—C1	1.2893 (17)	C12—H12B	0.9900
O2—C2	1.2713 (17)	C13—C14	1.526 (2)
O3—C4	1.2579 (17)	C13—H13A	0.9900
O4—C5	1.2322 (17)	C13—H13B	0.9900
O5—C7	1.417 (2)	C14—C15	1.525 (2)
O5—H1	0.88 (3)	C14—H14A	0.9900
N1—C9	1.5005 (19)	C14—H14B	0.9900
N1—H2	0.90 (2)	C15—C16	1.525 (2)
N1—H3	0.89 (2)	C15—H15A	0.9900
N1—H4	0.87 (2)	C15—H15B	0.9900
C1—C6	1.370 (2)	C16—C17	1.525 (2)
C1—C2	1.5242 (19)	C16—H16A	0.9900
C2—C3	1.3950 (19)	C16—H16B	0.9900
C3—C4	1.3922 (19)	C17—C18	1.521 (2)
C4—C5	1.5517 (19)	C17—H17A	0.9900
C5—C6	1.4241 (19)	C17—H17B	0.9900
C7—C8	1.499 (2)	C18—C19	1.524 (2)
C7—H7A	0.9900	C18—H18A	0.9900
C7—H7B	0.9900	C18—H18B	0.9900
C8—H8A	0.9800	C19—C20	1.518 (3)
C8—H8B	0.9800	C19—H19A	0.9900
C8—H8C	0.9800	C19—H19B	0.9900
C9—C10	1.521 (2)	C20—H20A	0.9800
C9—H9A	0.9900	C20—H20B	0.9800
C9—H9B	0.9900	C20—H20C	0.9800
			
O1^i^—Cu1—O1	180.00 (6)	C11—C10—H10A	108.6
O1^i^—Cu1—O2^i^	84.09 (4)	C9—C10—H10B	108.6
O1—Cu1—O2^i^	95.91 (4)	C11—C10—H10B	108.6
O1^i^—Cu1—O2	95.91 (4)	H10A—C10—H10B	107.6
O1—Cu1—O2	84.09 (4)	C12—C11—C10	112.43 (12)
O2^i^—Cu1—O2	179.999 (1)	C12—C11—H11A	109.1
O1^i^—Cu1—O5^i^	94.24 (5)	C10—C11—H11A	109.1
O1—Cu1—O5^i^	85.76 (5)	C12—C11—H11B	109.1
O2^i^—Cu1—O5^i^	96.70 (4)	C10—C11—H11B	109.1
O2—Cu1—O5^i^	83.30 (4)	H11A—C11—H11B	107.9
O1^i^—Cu1—O5	85.76 (5)	C13—C12—C11	113.42 (12)
O1—Cu1—O5	94.24 (5)	C13—C12—H12A	108.9
O2^i^—Cu1—O5	83.30 (4)	C11—C12—H12A	108.9
O2—Cu1—O5	96.70 (4)	C13—C12—H12B	108.9
O5^i^—Cu1—O5	179.999 (1)	C11—C12—H12B	108.9
C1—O1—Cu1	112.48 (9)	H12A—C12—H12B	107.7
C2—O2—Cu1	112.47 (9)	C12—C13—C14	113.58 (13)
C7—O5—Cu1	136.61 (11)	C12—C13—H13A	108.9
C7—O5—H1	108.8 (16)	C14—C13—H13A	108.9
Cu1—O5—H1	110.5 (15)	C12—C13—H13B	108.9
C9—N1—H2	111.6 (12)	C14—C13—H13B	108.9
C9—N1—H3	111.9 (12)	H13A—C13—H13B	107.7
H2—N1—H3	106.3 (17)	C15—C14—C13	113.12 (13)
C9—N1—H4	110.2 (13)	C15—C14—H14A	109.0
H2—N1—H4	109.3 (17)	C13—C14—H14A	109.0
H3—N1—H4	107.4 (17)	C15—C14—H14B	109.0
O1—C1—C6	125.59 (13)	C13—C14—H14B	109.0
O1—C1—C2	115.10 (12)	H14A—C14—H14B	107.8
C6—C1—C2	119.31 (12)	C14—C15—C16	114.58 (13)
O2—C2—C3	124.12 (13)	C14—C15—H15A	108.6
O2—C2—C1	115.58 (12)	C16—C15—H15A	108.6
C3—C2—C1	120.29 (12)	C14—C15—H15B	108.6
C4—C3—C2	121.21 (13)	C16—C15—H15B	108.6
C4—C3—Cl2	119.01 (10)	H15A—C15—H15B	107.6
C2—C3—Cl2	119.74 (11)	C15—C16—C17	113.00 (13)
O3—C4—C3	125.49 (13)	C15—C16—H16A	109.0
O3—C4—C5	115.72 (12)	C17—C16—H16A	109.0
C3—C4—C5	118.79 (12)	C15—C16—H16B	109.0
O4—C5—C6	124.97 (13)	C17—C16—H16B	109.0
O4—C5—C4	116.59 (12)	H16A—C16—H16B	107.8
C6—C5—C4	118.43 (12)	C18—C17—C16	115.02 (13)
C1—C6—C5	121.74 (12)	C18—C17—H17A	108.5
C1—C6—Cl1	120.25 (11)	C16—C17—H17A	108.5
C5—C6—Cl1	117.98 (10)	C18—C17—H17B	108.5
O5—C7—C8	110.19 (15)	C16—C17—H17B	108.5
O5—C7—H7A	109.6	H17A—C17—H17B	107.5
C8—C7—H7A	109.6	C17—C18—C19	112.76 (14)
O5—C7—H7B	109.6	C17—C18—H18A	109.0
C8—C7—H7B	109.6	C19—C18—H18A	109.0
H7A—C7—H7B	108.1	C17—C18—H18B	109.0
C7—C8—H8A	109.5	C19—C18—H18B	109.0
C7—C8—H8B	109.5	H18A—C18—H18B	107.8
H8A—C8—H8B	109.5	C20—C19—C18	114.12 (15)
C7—C8—H8C	109.5	C20—C19—H19A	108.7
H8A—C8—H8C	109.5	C18—C19—H19A	108.7
H8B—C8—H8C	109.5	C20—C19—H19B	108.7
N1—C9—C10	111.53 (12)	C18—C19—H19B	108.7
N1—C9—H9A	109.3	H19A—C19—H19B	107.6
C10—C9—H9A	109.3	C19—C20—H20A	109.5
N1—C9—H9B	109.3	C19—C20—H20B	109.5
C10—C9—H9B	109.3	H20A—C20—H20B	109.5
H9A—C9—H9B	108.0	C19—C20—H20C	109.5
C9—C10—C11	114.68 (12)	H20A—C20—H20C	109.5
C9—C10—H10A	108.6	H20B—C20—H20C	109.5
			
O2^i^—Cu1—O1—C1	175.55 (10)	C2—C3—C4—C5	−2.5 (2)
O2—Cu1—O1—C1	−4.45 (10)	Cl2—C3—C4—C5	179.98 (9)
O5^i^—Cu1—O1—C1	79.23 (10)	O3—C4—C5—O4	0.18 (18)
O5—Cu1—O1—C1	−100.77 (10)	C3—C4—C5—O4	−179.84 (13)
O1^i^—Cu1—O2—C2	−178.08 (10)	O3—C4—C5—C6	179.43 (12)
O1—Cu1—O2—C2	1.92 (10)	C3—C4—C5—C6	−0.59 (19)
O5^i^—Cu1—O2—C2	−84.49 (10)	O1—C1—C6—C5	−177.45 (13)
O5—Cu1—O2—C2	95.51 (10)	C2—C1—C6—C5	2.4 (2)
O1^i^—Cu1—O5—C7	−104.22 (16)	O1—C1—C6—Cl1	0.2 (2)
O1—Cu1—O5—C7	75.77 (16)	C2—C1—C6—Cl1	−179.89 (10)
O2^i^—Cu1—O5—C7	171.24 (16)	O4—C5—C6—C1	179.66 (14)
O2—Cu1—O5—C7	−8.76 (16)	C4—C5—C6—C1	0.5 (2)
Cu1—O1—C1—C6	−174.25 (12)	O4—C5—C6—Cl1	1.9 (2)
Cu1—O1—C1—C2	5.85 (15)	C4—C5—C6—Cl1	−177.24 (9)
Cu1—O2—C2—C3	−178.16 (11)	Cu1—O5—C7—C8	39.7 (2)
Cu1—O2—C2—C1	0.64 (15)	N1—C9—C10—C11	−70.83 (16)
O1—C1—C2—O2	−4.48 (18)	C9—C10—C11—C12	177.58 (12)
C6—C1—C2—O2	175.61 (13)	C10—C11—C12—C13	−179.36 (12)
O1—C1—C2—C3	174.36 (13)	C11—C12—C13—C14	177.86 (13)
C6—C1—C2—C3	−5.5 (2)	C12—C13—C14—C15	−179.44 (13)
O2—C2—C3—C4	−175.74 (13)	C13—C14—C15—C16	174.36 (14)
C1—C2—C3—C4	5.5 (2)	C14—C15—C16—C17	176.68 (14)
O2—C2—C3—Cl2	1.7 (2)	C15—C16—C17—C18	176.30 (14)
C1—C2—C3—Cl2	−176.99 (10)	C16—C17—C18—C19	178.04 (15)
C2—C3—C4—O3	177.46 (13)	C17—C18—C19—C20	174.00 (16)
Cl2—C3—C4—O3	0.0 (2)		

Symmetry code: (i) −*x*+1, −*y*+1, −*z*+2.

### Hydrogen-bond geometry (Å, º)

**Table:**

*D*—H···*A*	*D*—H	H···*A*	*D*···*A*	*D*—H···*A*
O5—H1···O3^ii^	0.88 (3)	1.94 (3)	2.8145 (16)	172 (3)
N1—H2···O1^iii^	0.91 (3)	1.97 (2)	2.8562 (16)	167 (3)
N1—H3···O3	0.89 (3)	2.05 (3)	2.928 (2)	168.3 (17)
N1—H4···O3^iv^	0.87 (3)	2.12 (3)	2.9784 (19)	171 (3)
N1—H4···O4^iv^	0.87 (3)	2.50 (3)	2.9842 (19)	116.2 (13)

Symmetry codes: (ii) *x*+1, *y*, *z*; (iii) −*x*+1, −*y*, −*z*+2; (iv) −*x*, −*y*, −*z*+2.
